# Increase in outbreaks of gastroenteritis linked to bathing water in Finland in summer 2014

**DOI:** 10.2807/1560-7917.ES.2017.22.8.30470

**Published:** 2017-02-23

**Authors:** Ari Kauppinen, Haider Al-Hello, Outi Zacheus, Jaana Kilponen, Leena Maunula, Sari Huusko, Maija Lappalainen, Ilkka Miettinen, Soile Blomqvist, Ruska Rimhanen-Finne

**Affiliations:** 1 National Institute for Health and Welfare, Department of Health Security, Water and Health Unit, Kuopio, Finland; 2 National Institute for Health and Welfare, Department of Health Security, Viral Infections Unit, Helsinki, Finland; 3 National Supervisory Authority for Welfare and Health, Helsinki, Finland; 4 University of Helsinki, Faculty of Veterinary Medicine, Department of Food Hygiene and Environmental Health, Helsinki, Finland; 5 National Institute for Health and Welfare, Department of Health Security, Infectious Disease Control Unit, Helsinki, Finland; 6 Helsinki University Hospital, Laboratory Services (HUSLAB), Department of Clinical Microbiology, Helsinki, Finland

**Keywords:** bathing water, waterborne outbreaks, surveillance, norovirus, gastrointestinal disease, faecal indicator bacteria

## Abstract

An increased number of suspected outbreaks of gastroenteritis linked to bathing water were reported to the Finnish food- and waterborne outbreak (FWO) registry in July and August 2014. The investigation reports were assessed by a national outbreak investigation panel. Eight confirmed outbreaks were identified among the 15 suspected outbreaks linked to bathing water that had been reported to the FWO registry. According to the outbreak investigation reports, 1,453 persons fell ill during these outbreaks. Epidemiological and microbiological data revealed noroviruses as the main causative agents. During the outbreaks, exceptionally warm weather had boosted the use of beaches. Six of eight outbreaks occurred at small lakes; for those, the investigation strongly suggested that the beach users were the source of contamination. In one of those eight outbreaks, an external source of contamination was identified and elevated levels of faecal indicator bacteria (FIB) were noted in water. In the remaining outbreaks, FIB analyses were insufficient to describe the hygienic quality of the water. Restrictions against bathing proved effective in controlling the outbreaks. In spring 2015, the National Institute for Health and Welfare (THL) and the National Supervisory Authority for Welfare and Health (Valvira) published guidelines for outbreak control to prevent bathing water outbreaks.

## Introduction

Since 1997, municipal authorities in Finland have been submitting notifications on suspected outbreaks of illness caused by drinking water to a national food- and waterborne outbreak (FWO) registry online, developed and maintained by the National Institute for Health and Welfare (THL) and the Finnish Food Safety Authority (Evira) [1]. Notification is mandatory and involves outbreaks with more than five cases who are not family members, have similar symptoms and have been exposed to the same water in a particular time period. The purpose of the FWO registry is to promote surveillance and outbreak investigations and to facilitate reporting at national and international level. During the period from 2009 to 2013, between one and eight waterborne outbreak notifications were sent to the FWO registry each year. Since 2012, the FWO registry has also been receiving notifications on suspected outbreaks caused by bathing water [2].

In Finland, the general provisions governing water quality at public beaches are included in the Health Protection Act (763/1994). More specific provisions concerning the monitoring and management of water quality at large and small public beaches (≥ 100 and < 100 bathers in a day, respectively) are included in the Decrees of the Ministry of the Social Affairs and Health (177/2008 and 354/2008) [3,4]. Legislation for large public beaches is based on the European Union’s Bathing Water Directive (BWD) 2006/7/EC and includes a requirement for the classification of bathing waters based on frequent monitoring during the last four bathing seasons [5]. The BWD also requires beach owners to inform the public about bathing water quality and beach management, through signs at the beaches and via the Internet. Furthermore, bathing water profiles are used for public information. These profiles contain, for instance, information on the sources of pollution that may affect the quality of the bathing water and are a risk to bathers' health.

The quality of bathing waters is assessed according to the monitoring results of two faecal indicator bacteria (FIB), *Escherichia coli* and intestinal enterococci. In the Finnish legislation, the microbiological values for management actions have been set separately for *E. coli* and intestinal enterococci [3,4]. For inland bathing waters, these values are 1,000 and 400 colony-forming units (CFU) or most probable number (MPN) per 100 mL, and for coastal bathing waters, 500 and 200 CFU or MPN per 100 mL for *E. coli* and intestinal enterococci, respectively. If these values are exceeded, the municipal health protection authority has to assess the impact of the deteriorated water quality on bathers’ health. The authority may give instructions or impose restrictions such as advice against bathing or a temporary bathing prohibition so as to prevent health hazards. Bathing water on large public beaches is classified as excellent, good, sufficient or poor [5]. The higher the concentration of indicator bacteria and their standard deviation in bathing water, the lower is the status of the bathing water. In excellent bathing waters, the concentrations of *E. coli* and intestinal enterococci are very low, indicating no source of faecal pollution.

It has been estimated that globally ca 120 million cases of gastrointestinal disease and ca 50 million cases of respiratory diseases are caused by swimming in wastewater-polluted waters each year [6]. Thus, the health risks related to bathing water have been commonly recognised and several pathogenic microbes are known to spread via water. Viruses have caused an increasing number waterborne outbreaks associated with recreational activities [7-10] and according to a survey of 55 viral outbreaks, noroviruses were with 45% the most prevalent causative agent [11].

In July 2014, THL received primary information on several suspected outbreaks linked to bathing water via the media, while no notifications were reported to the FWO registry. This resulted in direct contacts with the health authorities, and a reminder about notifying outbreaks related to bathing water was posted in a THL Infectious Disease Bulletin sent to the municipal health authorities. The message was also distributed to municipal environmental authorities by the National Supervisory Authority for Welfare and Health (Valvira). Following these reminders, several notifications were reported to the FWO registry. We identified outbreaks caused by bathing water from the FWO registry for 2014 and reviewed the epidemiological and microbiological data in order to assess and compile guidelines for outbreak control to prevent similar outbreaks in the future.

## Methods

### Epidemiological investigation

We reviewed outbreak notifications and investigation reports from the FWO registry for 2014. Outbreaks with a suspected link to bathing water were included in this study. We evaluated the strength of association for waterborne outbreaks based on classification criteria (Table 1) modified from those presented by Tillett et al. [12] and on information collected from local investigation reports (i.e. time and place of swimming, number of ill persons, clinical and microbiological findings).

**Table 1:** Classification criteria used for evaluating the strength of association for waterborne outbreaks, Finland, 2014

A: Same pathogen identified in patients and in the environment	B: Water quality failure or other deviation in the quality of environment
C: Association between illness and environment shown in analytical epidemiological investigation	D: Descriptive epidemiological investigation suggests that the outbreak is related to the environment and excludes other obvious exposures

Strong association: A + C or A + D or B + C. Probable association: B + D or C or A. Possible association: B or D.

Criteria modified from Tillett et al. [12].

### Microbiological investigation

#### Description of the laboratories and their roles

Analyses of enteric virus were carried out in four laboratories. Clinical samples were analysed at the Helsinki University Hospital (HUSLAB) and/or at the Viral Infection Unit of the National Institute for Health and Welfare (THL). Water samples were analysed either at the Water and Health Unit of the National Institute for Health and Welfare (THL) or at the Department of Food Hygiene and Environmental Health, University of Helsinki (UH). Surface samples were analysed at the UH. Pathogenic bacteria, faecal indicator bacteria (FIB) and water temperature analyses were conducted in local clinical and/or environmental laboratories.

#### Clinical samples

Viruses were analysed in patients’ stools for seven outbreaks. At the HUSLAB laboratory, noroviruses were analysed according to Kanerva et al. [13]. For astroviruses, viral RNA was extracted from a 10% suspension of the stool using MagNa Pure LC (Roche, Germany). After RT-PCR, the amplified DNA was detected by liquid hybridisation using an astrovirus-specific probe [14]. At the THL laboratory, norovirus RNAs were extracted using the RNeasy Mini Kit (Qiagen, Germany) and the polymerase/capsid gene junction was amplified as previously described [14]. Genotyping analysis was done for several norovirus isolates at the THL laboratory. Viral RNA was amplified in polymerase region A using a one-step RT-PCR kit (Qiagen) according to Vinjé et al. [15]. Sequences were analysed using Geneious software. NoroNet online software was used for genotyping. For three outbreaks, stool specimens were tested for pathogenic bacteria (*Campylobacter,Salmonella*, *Shigella* and *Yersinia*) by routine methods [16].

#### Water samples

At the THL laboratory, noroviruses and adenoviruses were concentrated from 0.5–2 L water samples as previously described [17] and using glass fibre prefilters (Millipore). Viral nucleic acids were extracted and detected using RT-qPCR and qPCR methods, as previously described [18,19], with the exception of using Taqman Environmental Master Mix 2.0 (Life Technologies) in the adenovirus qPCR.

At the UH laboratory, noroviruses and adenoviruses were concentrated by using membrane disks HA and Nanoceram to filter a total volume of 4.5 L of water. When necessary, a prefilter (Waterra) was used, otherwise the protocol was as described in Maunula et al. [14]. As a modification, Taqman primer–probe sets were applied as published in ISO/TS 15216–2 [20] for norovirus GI and GII. Mengovirus was added as a process control.

MPN of *E. coli* and CFU of intestinal enterococci were analysed according to standards ISO 9308–2 and ISO 7899–2, respectively [21,22].

#### Surface samples

In outbreak IV, 10 environmental swabs were taken from the toilet facilities (toilets for females, toilets for males and two latrines). Swabs taken from taps, door handles and toilet seats were analysed for noroviruses according to Rönnqvist et al. using nucleic acid detection by RT-qPCR [23]. For adenovirus investigation, a primer–probe set from Jothikumar et al. was included [24].

### Statistical analyses

The statistical analyses were conducted using SPSS 22 software for Windows. The related samples Wilcoxon signed-rank test was used to test the significance of temperature and FIB analyses, while comparing the outbreak samples with frequent-monitoring samples collected during the summer. Differences were considered significant if the p value was < 0.05.

## Results

### Review of the outbreak notifications and investigation reports

In 2014, 15 outbreaks suspected to be caused by bathing water were reported to the FWO registry. We identified eight outbreaks in which an association between bathing water and the illness could be confirmed based on classification criteria (Table 1). These outbreaks occurred on public beaches in different parts of Finland in July and August, 2014 (Table 2; Table 3).

**Table 2:** Description of beaches with outbreaks linked to recreational water, Finland, summer 2014 (n = 13)

Outbreak	Type	Size (ha)	Category	Estimated number of bathers/day	EU BWD classification (2014)^a^	Estimated outbreak start time	Restriction against bathing
I	Lake	2,420	Small	< 100	NA	26 July	1–6 August
II	Lake	2.9	Large	150–500	Excellent	25 July	29 July–21 August
III^b^	Lake	5.5–141	2/6 small 4/6 large	< 100 > 100	NA Excellent	24–27 July	28 July–12 August
IV	Lake	16.6	Large	100–2,000	Excellent	24 July	31 July–31 August (until the end of the bathing season)
V	Lake	9.7	Small	< 100	NA	3 August	15–22 August
VI	Lake	71.1	Large	≤ 150	Excellent	5 August	11–21 August
VII	Sea	393,00,000	Small	< 100	NA	NK	13–15 August and 19 August–9 September
VIII	Lake/pond	0.8	Large	1,000	NA^c^	27 July	6–21 August

EU BWD: European Union’s Bathing Water Directive [5]; NA: not available; NK: not known.

^a^ Based on frequent monitoring during the last four bathing seasons [5].

^b^ Combined results from six beaches.

^c^ New beach, no classification.

**Table 3:** Strength of association for waterborne outbreaks, number of patients, virological findings and observed quality deviations, Finland, summer 2014 (n = 1,453 patients)

Outbreak	Strength of association^a^	No. of patients	Viruses found in patients	No. of virus findings per water samples tested	Viruses found in water	Observed quality deviation
I	Possible (D)	40	NA	0/1	ND	Not observed
II	Probable (A + B)	85	Norovirus GI.2	2/4	Adenovirus, norovirus GI	Untidy toilets
III^b^	Strong (B + C)	819^b^ 1,093^c^	Norovirus GI.2, GI.4, GII.2	0/3	ND	Untidy toilets, defecation in water
IV	Strong (A + B + D)	185	Norovirus GII	0/1	ND	Untidy toilets
V	Probable (A)	4	Norovirus GI.2 and GII.4	1/2	Norovirus GII	Not observed
VI	Possible (B)	17	Norovirus (not typed)	0/2	ND	Untidy toilets, used nappies in water
VII	Possible (B)	2	Norovirus GI	NA	NA	Wastewater overflow
VIII	Possible (B)	27	Astrovirus	1/3	Adenovirus	Faeces on the dock

NA: not analysed; ND: not detected.

^a^ Letters refer to classification criteria detailed in Table 1.

^b^ Combined results from six beaches that were investigated in detail.

^c^ Total number from all 32 suspected beaches from which the local health authority received notifications of illness.

Six of eight confirmed outbreaks occurred at rather small lakes or ponds (< 141 ha) and eight of 13 beaches were categorised as large public beaches with more than 100 bathers per day (Table 2). According to the BWD classification criteria based on the last four bathing seasons, all these large public beaches were classified as excellent, except for one beach that was opened in 2012 and therefore did not have data for classification.

Restrictions against bathing were set for each beach (Table 2). The length of these restrictions varied from 2 days to more than 3 weeks and for one beach, the advice against bathing was set for the rest of the bathing season. Seven of eight outbreaks occurred at inland lakes where no clear source of contamination was identified according to the bathing water profiles and/or outbreak investigation reports, although for five of these outbreaks at inland lakes, non-specific quality deviations were reported (Table 3). In the one coastal sea water outbreak, a wastewater overflow was identified as a potential source of contamination.

According to the outbreak investigation reports, 1,453 persons fell ill in these outbreaks (Table 3). The most common symptoms were vomiting, diarrhoea, stomach pain, and fever. Information on the incubation period was available for four outbreaks, the median incubation period ranging from 20 to 62 hours. The duration of illness was reported for five outbreaks, with a median ranging from 19 to 60 hours. None of the patients required hospital care.

### Microbiological findings

Patient samples were collected in seven outbreaks and tested for gastrointestinal pathogenic viruses and bacteria. Several types of norovirus were identified, with norovirus GI.2 detected in three outbreaks (Table 3). In addition, norovirus GI.4, GII.2 and GII.4 were detected in patient samples. In one patient, astrovirus was identified. According to outbreak investigation reports, pathogenic bacteria were analysed in three investigations (outbreaks III, IV and VIII). *Campylobacter* was found in one patient (outbreak III). *Salmonella*, *Shigella* or *Yersinia* spp. were not found in any of the specimens tested.

Water samples were collected for noro- and adenovirus analyses in seven outbreaks, and noro- and/or adenoviruses were detected in the samples from three outbreaks (Table 3). In the remaining outbreak, these analyses were not requested by the municipal health protection authority. FIB were analysed from water in all outbreaks. In addition, water quality monitoring was carried out at every beach according to EU BWD and national regulations. Elevated levels of both FIB were found in two of the outbreaks (VII and VIII; Table 4), but only in outbreak VII did the number of *E. coli* exceed the limit for management actions, with maximum concentrations of 1,100 and 190 CFU/100 mL for *E. coli* and enterococci, respectively. Elevated levels of enterococci were also noted in outbreak I. In the remaining outbreaks, the levels of FIB were low. Overall, no statistical difference in the levels of *E. coli* (p = 0.8) or enterococci (p = 0.086) were noted between the outbreak samples (n = 14) and the frequent-monitoring samples (n = 42), excluding the samples from outbreak VII, where a clear contamination source was noted.

**Table 4:** Levels of faecal indicator bacteria and water temperature in outbreak samples (n = 17) and frequent-monitoring samples (n = 47), Finland, summer 2014

Outbreak	No. of analysed water samples	*Escherichia coli* MPN/100 mL	Intestinal enterococci CFU/100 mL	Temperature °C
I Outbreak samples Monitoring samples	1 3	6 8 ± 6	190 4 ± 2	25.7 22.1 ± 3.3
II Outbreak samples Monitoring samples	2 6	39 ± 26 72 ± 72	9 ± 8 6 ± 4	25.0 ± 1.4 20.5 ± 4.4
III^a^ Outbreak samples Monitoring samples	5 18	14 ± 10 19 ± 4	3 ± 3 15 ± 22	25.2 ± 0 19.0 ± 3.8
IV Outbreak samples Monitoring samples	1 4	9 3 ± 3	7 1 ± 2	24.0 19.8 ± 4.2
V Outbreak samples Monitoring samples	1 2	12 34 ± 47	22 6 ± 8	24.0 19.3 ± 2.5
VI Outbreak samples Monitoring samples	2 4	4 ± 1 1 ± 0	3 ± 2 1 ± 1	23.0 ± 0 17.5 ± 3.7
VII Outbreak samples Monitoring samples	3 5	670 ± 580 2 ± 4	110 ± 98 4 ± 4	22.3 ± 1^b^ 20.2 ± 3.5
VIII Outbreak samples Monitoring samples	2 5	130 ± 120 17 ± 5	48 ± 46 8 ± 7	23.9 ± 1 18.1 ± 2.2

CFU: colony-forming units; MPN: most probable number.

^a^ Combined results from the five beaches for which indicator bacteria were analysed.

^b^ Average from n = 2 samples.

At one outbreak (IV), 10 surface samples from the toilet area were analysed, and norovirus GII was found on the tap of the women’s toilet. Adenoviruses were not detected in the surface samples.

### Water temperature

During the outbreak period, exceptionally warm weather raised the temperature of the bathing water by several degrees (Table 4). The average temperature of the bathing water samples collected during the outbreaks was 24.3 ± 1.3 °C (n = 16), while the average temperature of other frequent-monitoring samples collected at these beaches in summer 2014 (2 June to 26 August) was 19.4 ± 3.6 °C (n = 47; p = 0.002).

## Discussion

In 2014, an increased number of suspected outbreaks linked to bathing water were reported to the Finnish FWO registry. Reminders about the need to notify outbreaks borne by bathing water were sent to the municipal authorities and probably triggered the following notifications seeing as only one outbreak linked to bathing water had been reported during the period 2012 to 2013. In addition, the publicity around outbreaks in 2014 probably made the beach users’ more alert so that they reported their suspicions of bathing water-related sickness to the health authorities. Generally, it could be difficult to attribute individually reported gastroenteritis cases to a particular bathing activity and therefore these outbreaks may remain undocumented.

Nearly 1,500 persons fell ill during the outbreaks linked to bathing water in 2014. Although the exact number of people visiting the beaches was not known, some municipal investigation reports estimated that hundreds to thousands of persons per day had been swimming at each beach during the outbreak period before restrictions against bathing were set. In the summer of 2014, the period of continuous hot weather in Finland, with temperatures of more than 25 °C, was exceptionally long and lasted for 38 days [25]. Because of this heatwave, it is likely that more people than usual were visiting the beaches and spent more time in the water. A previous study noted a positive correlation between the number of days with temperatures over 25 °C and the number of outbreaks per bathing season [26]. Some investigation reports also stated that the toilets at the beaches were untidy, rubbish bins were overloaded, and used nappies were floating in the water, indicating overcrowded conditions. In 2015, no outbreaks linked to bathing water were reported. This was probably due in part to the weather conditions, namely 3 days with temperatures over 25 °C in July 2015, compared with 26 such days in July 2014. In Helsinki, the average temperature and precipitation in July differed considerably between 2015 and 2014 (16.2 °C/76.1 mm vs 20 °C/12.5 mm) [27].

Most of the beaches were small, suggesting that the volume of users exceeded the self-cleaning capacity of the beach. For example, the volume of the smallest lake (outbreak VIII) is 20,800 m^3^. In theory, if a single infected person excreted large numbers of noroviruses (up to 10^11^ genomic copies/g) [28], and if these viruses were evenly diluted in the total volume of the lake, 1 g of faeces would result in a virus concentration of nearly 5,000 genomic copies/L. Considering the low infectious dose of norovirus (as few as 18 virus particles) [29] and the average ingestion of water while swimming (37 mL and 16 mL for children and adults, respectively, per 45 min swimming session [30]), it is obvious that the bathing water at this particular beach would have the potential to cause a considerable number of infections.

Norovirus was detected in ill persons in most of the outbreaks. The symptoms reported by municipal authorities fit the clinical picture of a norovirus illness [31]. In three outbreaks, norovirus GI.2 was identified. In addition, also GI.4, GII.2 and GII.4 were detected in patient samples. The prevalence of GI in these outbreaks is consistent with the observation that GI genotypes are more frequently involved in food- or waterborne outbreaks than GII, which could imply that GI is more stable in the environment [32,33]. Genotype GII.4 is the most common genotype causing infections in humans and is more likely to be associated with person-to-person transmission [34].

In two outbreaks, norovirus GI and GII were found in bathing water and in one outbreak, GII was determined in a swab taken from the tap of the toilet, but the number of particles obtained was too small to allow typing of these viruses. Therefore, an exact comparison between patient and water samples could not be carried out. In two outbreaks, adenovirus was found in water. Adenoviruses are commonly found in human wastewater and owing to their high stability in aqueous environments, they are recognised as good viral indicators of human sewage pollution [19,35,36]. Moreover, adenoviruses can spread via contaminated water and they have been linked to waterborne outbreaks [14,37,38]. Since adenoviruses most often result in subclinical disease, and symptomatic infections tend to be mild and self-resolving, most infections remain undocumented [39]. In the outbreaks of this study, no adenoviruses were identified in ill persons.

In Finland, the hygienic quality of the bathing water is evaluated according to BWD and national regulations [3-5]. According to Finnish legislation, the minimum number of bathing water samples to be taken during a bathing season is three for small public beaches and four for large public beaches. The legislation contains rules how to monitor and manage bathing waters, indicates microbiological threshold values, regulates measures to be taken when bathing water fails to meet the quality and requires the dissemination of information about bathing water quality. In Finland, the concentrations of FIB in bathing water are typically very low; 70% of the *E. coli* and 58% of the intestinal enterococci concentrations were < 10 CFU or MPN/100 mL in bathing water samples collected from all large public beaches (n = 302) during the seasons from 2013 to 2015 (data not shown). In this study, the microbiological threshold for management actions was exceeded only in one of eight outbreaks. For this outbreak, a clear external contamination source was identified as 2,000–3,000 m^3^ of raw wastewater had overflowed near the bathing site. In the other outbreaks, the levels of FIB were low and the bathing water quality was classified as excellent according to the BWD criteria. The sources of contamination in these outbreaks were most probably the bathers and other beach users. This suggestion is supported by the observed pollution of the beach environment.

The poor indicator value of FIB in these outbreaks raises questions about the current practices for assessing bathing water quality. This finding is consistent with a recent study showing high prevalence of adenoviruses (75%) in bathing water samples, which nevertheless complied with the regulations for recreational use [40]. Moreover, Boehm et al. reviewed the lack of correlation between FIB and human pathogen concentrations and between FIB and human health, especially in recreational areas of non-point-source contamination [41]. It is also widely known that pathogenic microbes, especially enteric viruses, survive substantially better than the currently used FIB in water environments. Therefore, new candidates, such as *Clostridium perfringens*, coliphages, *Bacteroides* and human enteric viruses as well as new genomic approaches, e.g. metagenomics, have been proposed for water quality assessment [41-43]. However, during the summer, the higher temperature of bathing water and the increased amount of ultraviolet light have a negative impact on microbe survival. In this study, noro- and adenoviruses in outbreak II were detected in the water on at least six days but fewer than 12 days. These relatively short contamination episodes may remain undetected with routine FIB sampling. In most of the outbreaks, the quality of bathing water was questioned only after people visiting the beaches fell ill, and restrictions against bathing were set for the beaches only then. The length of the restrictions was determined according to the results of water analyses and proved effective in controlling of the outbreaks.

Investigation reports of outbreaks linked to bathing water were assessed by a panel that included experts from THL, Valvira and UH. By using agreed criteria, reports can be assessed more consistently over time [12]. When the same pathogen has been identified in patients and in the beach environment, results from the analytical epidemiological study point towards a certain source and water quality failures have been detected, outbreaks are often easy to categorise. More discussion in the panel will be needed on the relation between illness and the beach environment when pollution of the beach is mentioned but no obvious other exposures are described in outbreak reports. In this study, eight outbreaks were identified among the 15 outbreaks suspected to be caused by bathing water that were reported to the FWO registry. Four outbreaks were classified as having a strong or probable association with the beach environment, and four as having a possible association. Analytical epidemiological investigations were lacking in all but one investigation, indicating that more training and practical experience in analytical epidemiology may be needed in the municipal outbreak investigation groups.

Because of an increase in the number of bathing water outbreaks in the summer of 2014, THL and Valvira published guidelines for outbreak control in spring 2015 to prevent bathing water outbreaks. If, based on the laboratory or epidemiological findings, the water is considered to be contaminated, visitors should be informed about a bathing prohibition or advice against bathing should be posted by means of the international symbols presented in the Commission Implementing Decision (2011/321/EU) [44]. To prevent outbreaks, rooms intended for washing and dressing as well as toilets at the beach should be kept clean, and soap, hand towels and toilet paper should be available. Visitors should be encouraged to wash their hands or use freshen-up towels. Nappies should not be changed and the babies’ bottoms should not be washed in the bathing water, and people with gastrointestinal illness should avoid swimming. In the case of an outbreak suspicion, municipal authorities should notify the FWO registry and an outbreak investigation, including epidemiological and microbiological analyses, should be initiated.
